# Traumatic Obturator Hip Dislocation with Marginal Femoral Head Fracture in a 15-Year-Old Adolescent: A High-Energy Trauma—A Case Report and a Review of the Literature

**DOI:** 10.1155/2018/7268032

**Published:** 2018-07-16

**Authors:** Yoann Durand, Clémence Bruyère, Marco Saglini, Aurélien Michel-Traverso

**Affiliations:** ^1^Department of Orthopaedics and Traumatology Surgery, Riviera-Chablais Hospital, Monthey, Switzerland; ^2^Department of Radiology, University Hospital Geneva (HUG), Geneva, Switzerland; ^3^Cabinet de Chirurgie Orthopédique, Préverenges, Switzerland; ^4^Department of Orthopaedics and Traumatology Surgery, University Hospital of Lausanne (CHUV), Lausanne, Switzerland

## Abstract

We report the case of a 15-year-old boy brought to the emergency department after a bike accident, complaining of an isolated left hip pain. The X-rays showed an obturator hip dislocation treated by closed reduction under general anaesthesia, followed by 6 weeks of discharge. The follow-up MRI performed 6 weeks after the trauma showed an avascular femoral head necrosis, for which we performed multiple retrograde femoral head drilling, completed by the injection of autologue stem cells from the iliaq crest. One year later, the patient has no hip pain, no joint limitation, and can practice BMX at a high level again. The purpose of this report is to make the physicians aware of this rare problem that may be damaging for hip function, especially in young people.

## 1. Introduction

Traumatic obturator hip dislocation is rare in paediatric traumatology, and only a few cases have been reported in the English literature [1, 2]. In the adult population, an anterior-inferior dislocation is the least common of all the hip dislocations [3], counting only for 7% of the latter [4]. Furthermore, Stewart and Milford reported that hip dislocation is twenty five times less common in children than in adults [5]. Since the force required to cause hip dislocation increases with age in pediatric patients [6], a high-energy injury is needed to dissociate the femoral head from the acetabulum in teenagers. The main risk after hip dislocation is femoral head avascular necrosis [7]. Indeed, it occurs in 3 to 12 percent of patients [8–11], and it may be diagnosed until 24 months after the injury [12]. We report the case of a traumatic obturator foramen hip dislocation in a 15-year-old boy. We present radiological findings, orthopaedic follow-up, and clinical outcome.

## 2. Case Report

A 15-year-old boy was brought to the emergency department, presenting pain in his left hip after a bike accident during a BMX race. Physical examination showed an external rotation of the lower limb and an irreducible hip flexum. The patient was not able to move the hip nor bear weight. Additional examination showed no neurovascular damage.

**Figure 1:**
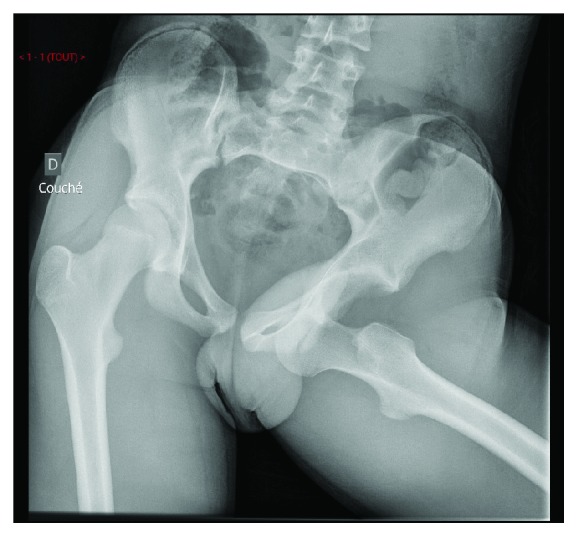
Emergency pelvic X-ray AP view.

**Figure 2:**
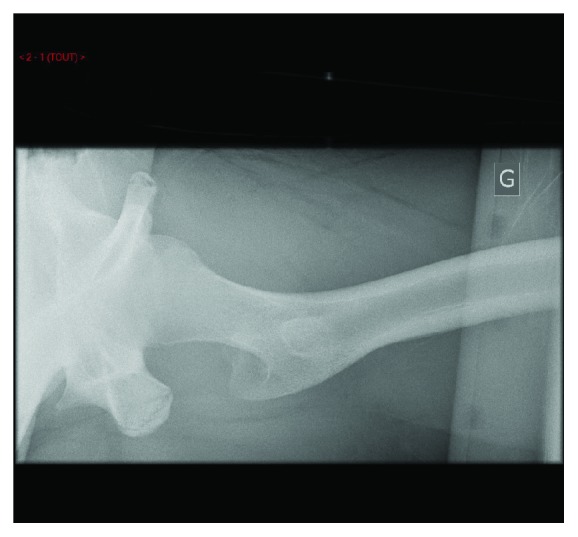
Emergency left hip X-ray axial view.

**Figure 3:**
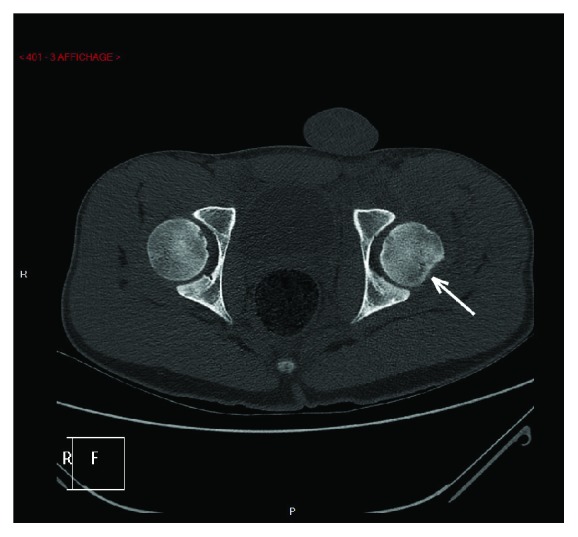
Hip CT-scan.

**Figure 4:**
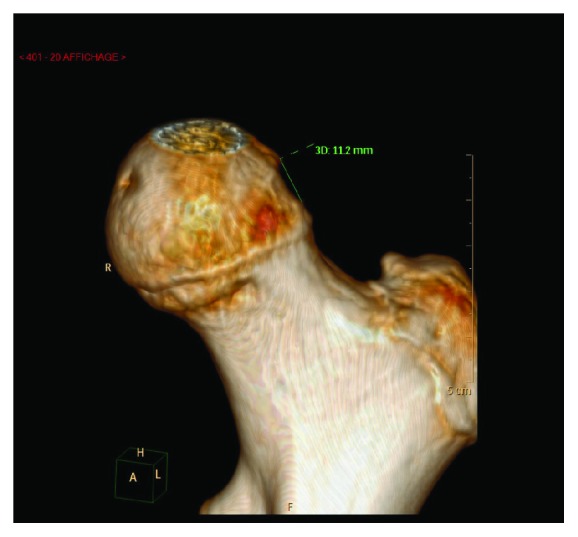
Hip CT-scan 3D view.

X-rays confirmed diagnosis of obturator hip dislocation (Figures 1 and 2). Closed hip dislocation reduction was immediately performed under general anaesthesia on an orthopaedic table. Like most hip dislocations in children, it resolved easily with gentle traction [13, 14]. The radiological assessment was completed with a CT scan, which showed a small impaction of the superolateral part of the femoral head (Figures 3 and 4), Pipkin classification type 1, and a small bone fragment in the obturator foramen. After the reduction, the patient was not allowed to bear weight for 6 weeks, as it is recommended for children older than 10 years [5], and hip flexion over 60 degrees was forbidden.

**Figure 5:**
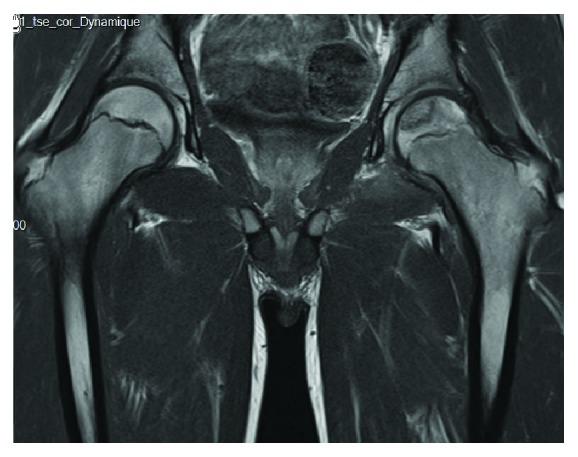
Hip MRI.

Gadolinium contrast MRI was realised 2 months after the trauma, diagnosing an internal and middle femoral head's pillar avascular necrosis (Figure 5), Steinberg classification type 1C.

**Figure 6:**
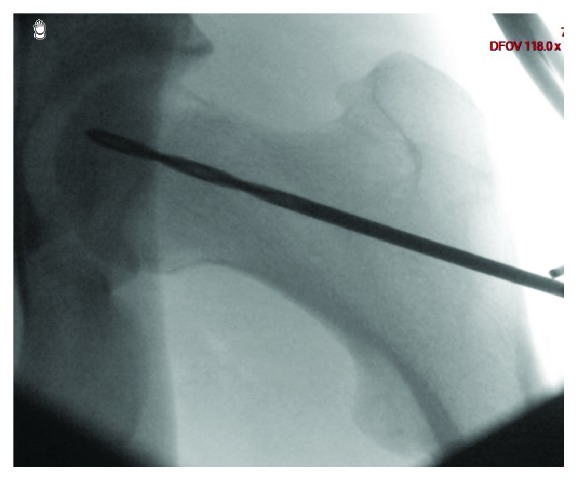
Left hip preoperative X-ray AP view.

We decided to perform a drilling of the femoral head followed by stem cell injection. Four boreholes were made from the greater trochanter up to the femoral head with a 3.2 mm drill (Figure 6), in which we placed autologue stem cells from the iliaque crest. After the operation, the patient was allowed to bear weight a maximum of 5 kilograms for 6 weeks.

On the 6th-week postoperative X-ray, we noticed a radiolucent area on the femoral head without loss of sphericity. We therefore performed an MRI 10 weeks after the drilling, which showed a slight depression of the superolateral angle of the femoral head, with resorption of the necrotic zone.

**Figure 7:**
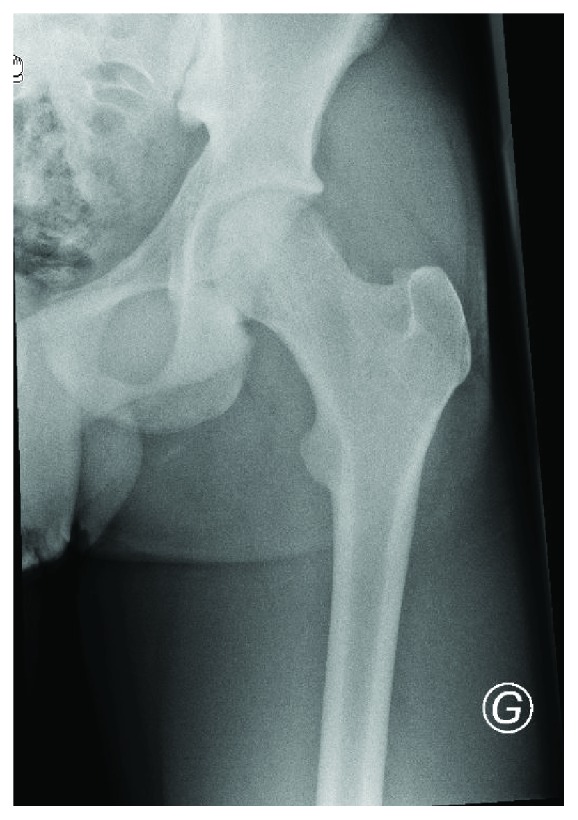
Left hip postoperative X-ray AP view.

One year after the surgery, the patient no longer complained of pain. He is able to walk without lameness and practice BMX at a high level again. Hip flexion is symmetric at 120 degrees. External and internal rotations were 30-0-20, versus 30-0-25 for the right hip. The X-rays (Figure 7) do not show any degenerative sign. Regarding social function and mobility, the patient scores were 1 on the assessment of Jensen (independent) and 9 on the assessment of Parker and Palmer (able to walk inside and outside the house and go shopping without any help). Both scores were the same compared to that before the trauma.

## 3. Discussion

Only a few cases of traumatic obturator hip dislocation in children have been reported in the English literature [1]. Although in young children hip dislocations usually result from mild trauma [7], in adolescent patients a high-energy trauma is required to dislocate the most stable joint in the body [6, 15]. Physical examination shows an irreducible hip flexion associated with an external rotation.

This injury must be reduced within 6 hours, since it has been shown that avascular necrosis was significantly associated with the dislocation duration [8]. We can therefore speculate that an initial ischaemia occurs while the hip is dislocated and causes avascular necrosis [16]. Nevertheless, this complication can occur even after an immediate reduction, especially in cases with a fracture of the femoral head, as we have seen here. Avascular femoral head necrosis occurs in an average of 9 percent of the cases [17] and can be shown by performing a gadolinium contrast MRI after the reduction.

In this case, we propose a surgical treatment with drilling of the femoral head and injection of stem cells from the iliaq crest, in order to preserve the native femoral head, especially in these young patients. As shown by Hernigou and Beaujean, core decompression with autologous bone marrow grafting from the iliac crest seems to improve the outcome of femoral head osteonecrosis [18]. For the patient whom we treated, follow-up, clinical, and radiological outcomes were conclusive, with an excellent hip mobility without any pain, one year after the surgery. However, we would need more patients with a long-term follow-up to prove the benefit of this innovative treatment.
